# Fetal intestinal dilatation and digestive tract abnormalities: an analysis of 94 cases from 16,090 deliveries in eastern Tianjin

**DOI:** 10.3389/fped.2026.1862935

**Published:** 2026-07-27

**Authors:** Shaohua Wang, Xinying Zhou, Ruifang Chen, Linxue Guan, Wenxiao Wen, Shanshan Zhao

**Affiliations:** The Third Central Hospital of Tianjin, Tianjin Key Laboratory of Extracorporeal Life Support for Critical Diseases, Artificial Cell Engineering Technology Research Centre, Tianjin Institute of Hepatobiliary Disease, Tianjin, China

**Keywords:** digestive system abnormalities, fetus, intestinal dilatation, isolated late-gestation, ultrasound

## Abstract

**Background:**

Prenatal detection of fetal intestinal dilatation (FID) presents significant counseling challenges, particularly in late gestation when the distinction between structural and functional causes guides clinical management. This study evaluated the diagnostic performance of prenatal ultrasound for distinguishing structural from functional gastrointestinal abnormalities in a large cohort with postnatally confirmed diagnoses.

**Methods:**

A retrospective analysis was conducted on 16,090 deliveries at a single tertiary center from January 2021 to May 2025. Ninety-four cases with prenatal FID were identified and followed postnatally. Diagnostic performance metrics (sensitivity, specificity, predictive values) were calculated, and ultrasound characteristics were compared between structural and functional cases.

**Results:**

Among 94 FID cases, postnatal follow-up confirmed 27 (28.7%) structural abnormalities and 67 (71.3%) functional disorders. Prenatal ultrasound showed moderate sensitivity (63.0%, 95% CI: 43.9–79.3%) but low specificity (24.2%, 95% CI: 13.9–38.2%) for detecting structural anomalies. Isolated FID in late gestation (≥33 weeks) could not reliably distinguish structural from functional causes, with comparable detection rates (63.0% vs. 76.1%, *P* = 0.148) and similar intestinal diameters (median 1.72 vs. 1.85 cm, *P* = 0.147) between groups. ROC curve analysis confirmed poor predictive value of intestinal diameter alone (AUC = 0.413).

**Conclusion:**

Isolated late-gestation FID has limited diagnostic specificity for structural gastrointestinal abnormalities, with high false-positive rates. These findings emphasize the need for cautious prenatal counseling and systematic postnatal evaluation. The proposed ultrasound assessment framework represents preliminary findings requiring prospective validation.

## 1 Introduction

With the continuous advancement of ultrasound technology, prenatal ultrasound screening for fetal developmental abnormalities, including those of the fetal intestines, has become increasingly accurate. Multiple studies have indicated that fetal intestinal dilatation may suggest a poor digestive system prognosis (1, 2). In clinical practice, it has been observed that some children with a history of fetal intestinal dilatation indeed exhibit abnormal development of the digestive system; however, some newborns with congenital structural abnormalities of the digestive system had no abnormal prenatal ultrasound findings. Thus, there is no clear consensus regarding the association between prenatal ultrasound-detected fetal intestinal dilatation and neonatal digestive tract structural abnormalities, nor regarding the differential diagnostic value of prenatal ultrasound for upper vs. lower gastrointestinal system (GIS) abnormalities.

Existing studies have preliminarily explored the diagnostic value of prenatal imaging for fetal intestinal dilatation. Early cohort studies confirmed that fetal intestinal dilatation is associated with adverse neonatal digestive outcomes (3), and subsequent research has further clarified the characteristic imaging manifestations of different types of digestive tract abnormalities, including the “double-bubble sign” of upper gastrointestinal obstruction (4), the ultrasound features of small bowel obstruction and ileal atresia (5, 6), and diagnostic clues for intestinal malrotation with volvulus (7).

Fetal magnetic resonance imaging has also been applied as an auxiliary diagnostic method to delineate the location and extent of intestinal obstruction (8).

However, there is still no unified consensus on the differential diagnostic value of isolated intestinal dilatation in late gestation for structural vs. functional abnormalities, and the difference in prenatal ultrasound detection performance between upper and lower gastrointestinal structural anomalies remains to be verified in large clinical cohorts. To address these gaps, we formulated the following research question (RQ) and hypotheses. The research question is whether prenatal ultrasound-detected fetal intestinal dilatation is associated with structural abnormalities of the neonatal digestive system, and whether the diagnostic performance of prenatal ultrasound differs between upper and lower gastrointestinal system (GIS) abnormalities. Hypothesis 1 (H1) states that fetal intestinal dilatation detected by prenatal ultrasound is positively associated with structural abnormalities of the neonatal digestive system. Hypothesis 2 (H2) proposes that prenatal ultrasound has higher detection rates for upper GIS structural abnormalities (annular pancreas, duodenal web) than for lower GIS structural abnormalities (congenital anal stenosis, megacolon). To test these hypotheses, this study retrospectively reviews and analyzes relevant cases from our hospital over an approximately 5-year period.

## 2 Methods

### 2.1 Information and methods

#### 2.1.1 The study cohort

2.1.1.1 The study cohort comprised a total of 94 cases with prenatal fetal intestinal dilatation, identified from 16,090 deliveries at our hospital between 1 January 2021 and 31 May 2025. Postnatally, 27 cases were confirmed to have structural digestive tract abnormalities, while 67 cases were diagnosed with functional digestive disorders. For cases involving functional disorders, perinatal factors such as preterm birth, low birth weight, small for gestational age, perinatal asphyxia, infection, and hypoglycaemia were excluded. The gestational ages ranged from 35⁺^6^ to 41⁺^4^ weeks, with a mean gestational age of 39⁺^3^ weeks.

2.1.1.2 The tests were performed using calibrated GE (USA) Voluson E10 and E8 colour Doppler ultrasound diagnostic equipment with 2–5 MHz two-dimensional convex array probes. All ultrasounds followed ISUOG guidelines (9).

Standardization included standard patient positioning (supine with slight left lateral tilt to avoid vena cava compression), consistent measurement protocol (intestinal diameter measured at the widest transverse section, perpendicular to the intestinal long axis, excluding intestinal walls), and quality control (weekly equipment calibration and monthly senior sonographer review of measurement techniques).

Five qualified sonographers (≥5 years prenatal ultrasound experience, Chinese Medical Ultrasound Society certification) performed the examinations. Two experienced sonographers (≥10 years) independently remeasured 30 randomly selected cases (31.9%) to assess inter-observer reliability, blinded to the original data. The intestinal diameter intraclass correlation coefficient (ICC) was 0.92 (95% CI: 0.86–0.96), indicating good agreement.

##### 2.1.1.2.1 Follow-up scan frequency

For routine low-risk cases (with no signs of fetal intestinal dilatation or other structural abnormalities), we followed ISUOG recommendations, scheduling one baseline third-trimester scan (32–34 weeks) to assess mid-late gestational intestinal development and one final scan (37–38 weeks) to confirm fetal abdominal anatomy before delivery. This more frequent monitoring tracked dynamic changes in intestinal diameter (e.g., progressive dilatation or stability) and quickly identified new structural signs (such as the “double-bubble sign” suggesting duodenal obstruction), allowing prenatal counseling and postnatal management plans to be adjusted.

#### 2.1.2 Sonographic criteria for structural vs. functional dilatation

We used ISUOG-aligned criteria to distinguish structural from functional intestinal dilatation, which is crucial for diagnostic performance analyses: structural abnormalities were defined by ultrasound anatomical signs, such as annular pancreas/duodenal web [the “double-bubble sign”: dilated stomach + proximal duodenum, connected via the pylorus (10)], intestinal atresia, and prenatal examination identified those with intestinal diameter over the criteria but no structural symptoms as “functional”; postnatal follow-up confirmed this.

### 2.2 Research methods and diagnostic criteria

Fetal growth metrics and structural evaluation, including intestinal dilatation location, internal diameter, and extent, were measured during the prenatal ultrasound. The “double-bubble sign” shows the pylorus connecting the gastric bubble in the left upper abdomen to the dilated proximal duodenum in the right (10).

The colon has haustral markings and is peripherally in the lower abdomen, whereas the small intestine is in the mid-abdomen. Intestinal dilatation was indicated by a small intestinal diameter above 7 mm or a colonic internal diameter over 18 mm (11). Polyhydramnios was identified when amniotic fluid index surpassed 250 mm. Umbilical cord anomalies include nuchal cord entanglement, torsion, prolapse, excessive length (>100 cm), short chord (<30 cm), and single umbilical artery. The placenta has morphological deformities such succenturiate, membranous, and annular placentas, improper implantation like placenta praevia and placenta accreta, and functional diseases including placental insufficiency and abruption.

#### 2.2.1 Sample size calculation

Primarily, the difference in prenatal ultrasonography detection rates between neonates with structural and functional digestive system disorders defined the sample size. Using G*Power 3.1 software (Heinrich-Heine-Universität Düsseldorf, Düsseldorf, Germany), we set *α* = 0.05 (two-tailed), power (1−β) = 0.80, and effect size (*f*) = 0.30 [moderate, consistent with previous studies on fetal intestinal dilatation and digestive tract abnormalities (2)]. The computation showed that 76 people (25 in structural abnormalities and 51 in functional problems) were needed to detect the expected group difference. The requisite sample size was surpassed in our final research cohort of 94 people (27 in structural abnormalities and 67 in functional problems).

#### 2.2.2 Power analysis

A *post-hoc* power analysis was performed using SPSS 26.0 software to verify the statistical power of the study. Based on the actual sample size (*n* = 27 for the structural abnormalities group; *n* = 67 for the functional disorders group), *α* = 0.05, and the observed effect size (*f* = 0.28) from the primary outcome data, the *post-hoc* power was calculated as 0.89. This value exceeds the conventional threshold of 0.80, confirming that the study had sufficient statistical power to detect meaningful differences between the two groups.

### 2.3 Statistical analysis

Statistical analyses were performed using SPSS 26.0 software. Continuous variables were expressed as mean ± standard deviation for normally distributed data (assessed by Shapiro–Wilk test) and compared using independent-samples *t*-tests. Non-normally distributed data (e.g., intestinal diameter) were expressed as median (interquartile range) and compared using the Mann–Whitney *U* test. Categorical variables were expressed as counts (percentages) and compared using the chi-square (*χ*^2^) test.

Primary analysis: A two-tailed *P* < 0.05 was considered statistically significant for the main between-group comparisons.

Exploratory subgroup analysis: For multiple comparisons in secondary analyses, the Benjamini-Hochberg false discovery rate correction was applied. Subgroup findings with small sample sizes (<10 cases per group) should be interpreted cautiously as hypothesis-generating only, requiring validation in larger cohorts.

### 2.4 Study flow chart

Following STROBE (Strengthening the Reporting of Observational Studies in Epidemiology) recommendations (12), a flow chart shows participant inclusion and exclusion (Supplementary Figure S1). Briefly:

Inclusion criteria: (1) Deliveries at The Third Central Hospital of Tianjin between 1 January 2021 and 31 May 2025; (2) Prenatal ultrasound records documenting fetal intestinal dilatation, or postnatal diagnosis of digestive tract structural abnormalities or functional digestive disorders; (3) Complete clinical data.

Fetal chromosomal or genetic abnormalities (confirmed by karyotype analysis or whole-exome sequencing); multiple gestations (excluding dichorionic diamniotic twins with independent digestive tract evaluations); severe maternal comorbidities (e.g., severe preeclampsia with organ failure, advanced liver/kidney disease) that may confound fetal intestinal development; and incomplete data.

Initially, 112 instances were suspected. After excluding 18 instances (6 with chromosomal abnormalities, 4 multiple gestations, 3 severe maternal comorbidities, 5 with missing data), 94 cases were included in the research.

## 3 Results

### 3.1 Clinical features and postnatal outcomes of prenatal fetal intestinal dilatation

Among the 16,090 deliveries at our hospital between January 2021 and May 2025, 94 cases presented with prenatal fetal intestinal dilatation, corresponding to an overall incidence of 0.58%. Postnatal follow-up confirmed 27 cases (28.7%) with structural digestive tract abnormalities and 67 cases (71.3%) with functional digestive disorders.

Among the 27 structural abnormalities, the disease distribution was as follows: congenital anal stenosis (*n* = 17, 63.0%), congenital anal atresia (*n* = 2, 7.4%), congenital megacolon (*n* = 3, 11.1%), intestinal atresia (*n* = 2, 7.4%), and rare upper gastrointestinal anomalies (*n* = 3, 11.1%), including 1 case of annular pancreas, 1 case of gastric duplication, and 1 case of duodenal web.

Prenatal intestinal ultrasound detected abnormal findings in 17 of the 27 cases (63.0%) with structural digestive tract abnormalities. The three upper gastrointestinal anomalies (gastric duplication, duodenal web, and annular pancreas, all presenting with a double-bubble sign) were clearly identified during prenatal screening at 24–36 weeks of gestation, and all were subsequently confirmed by postnatal surgery. The remaining prenatally detected structural cases presented with intestinal dilatation measuring 1.4–2.3 cm in width, identified at gestational ages of 35⁺^1^–40⁺^6^ weeks. Among the 10 structural cases with no abnormal prenatal ultrasound findings, six were postnatally diagnosed with congenital anal stenosis, two with congenital megacolon, one with congenital anal atresia, and one with intestinal obstruction—all representing lower digestive tract structural abnormalities.

The 67 functional digestive disorders comprised meconium plug syndrome (*n* = 3, 4.5%), gastroesophageal reflux (*n* = 6, 9.0%), and idiopathic transient dilatation (*n* = 58, 86.6%). Prenatal ultrasound detected intestinal dilatation (the primary abnormal finding) in 51 of these 67 cases (76.1%). All functional cases with abnormal ultrasound findings presented with isolated fetal intestinal dilatation measuring 1.5–2.8 cm in width, identified at late gestational ages (33⁺^2^–40⁺^6^ weeks, predominantly within 2 weeks before delivery).

The prenatal detection rate of intestinal dilatation was 63.0% (17/27) in the structural abnormality group and 76.1% (51/67) in the functional disorder group, with no statistically significant difference between the two groups (*χ*^2^ = 2.094, df = 1, *P* = 0.148) (Table 1).

**Table 1:** 2 × 2 Contingency table of prenatal ultrasound results vs. postnatal definitive diagnosis of structural digestive tract abnormalities.

Prenatal ultrasound result	Postnatal structural anomaly diagnosis	Postnatal structural anomaly diagnosis	Row total
	Positive (+)	Negative (−)	
Positive (+)	True positive (TP) = 17	False Positive (FP) = 51	17 + 51 = 68
Negative (−)	False negative (FN) = 10	True Negative (TN) = 16	10 + 16 = 26
Column total	17 + 10 = 27 (total structural anomalies)	51 + 16 = 67 (total non-structural cases)	Grand total = 68 + 26 = 94 cases

During the study period, the annual proportion of digestive system abnormalities detected by prenatal ultrasound relative to total deliveries showed a marked year-on-year increase: 0.04% (2/4,125) in 2021, 0.1% (5/4,816) in 2022, 0.1% (4/3,256) in 2023, 0.5% (20/3,481) in 2024, and 2% (35/1,700) from January to May 2025.

All functional disorders resolved with conservative management. For the 58 cases of idiopathic transient dilatation, the standardized conservative regimen included fasting combined with nasogastric tube gastrointestinal decompression, intravenous fluid replacement and parenteral nutrition, warm normal saline enema (1–2 times daily), oral probiotic supplementation, and gradual enteral feeding. All 58 cases achieved clinical recovery, with a mean recovery time of 4.8 ± 1.5 days (range: 3–7 days), defined as complete resolution of abdominal distension, restoration of regular bowel movements, and successful transition to full enteral feeding without intolerance. All cases were followed up for 6–12 months (median: 8 months) via postnatal outpatient assessments and abdominal ultrasound re-examinations; no recurrence of intestinal dilatation, feeding difficulties, or other digestive tract complications was reported, and all neonates exhibited age-appropriate growth and development.

3.2 There was no statistical significance in comparison between structural problems and functional problems in newborn digestive system. The mean values of the abnormal expansion of the intestinal tube were 1.76 + 265 and 1.88 + 240 cm respectively, and there was no statistical significance (*F* = 0.266, *t* = 1.539, *P* = 0.129) between the two groups.

Table 1 is a 2 × 2 contingency table designed to compare the prenatal ultrasound detection rates between neonates with postnatal structural digestive tract abnormalities and those with non-structural (functional) disorders. For analyzing categorical data in a 2 × 2 table, we employed the Pearson's Chi-square (*χ*^2^) test—the standard statistical method for assessing associations between two binary variables (here: “prenatal ultrasound result” [positive/negative] and “postnatal diagnosis” [structural anomaly/non-structural disorder]).

3.3 Maternal age, hypertension, diabetes, hypothyroidism, and the rate of non-Lactococcus colonisation showed no significant differences between the two groups of mothers of neonates with structural or functional digestive system abnormalities. Similarly, no significant differences were observed between the two groups in infant gender, placental abnormalities, birth order, or head circumference. However, significant differences were identified in birth weight, birth length, neutrophil count, lymphocyte count, haemoglobin level, platelet count, liver function, and calcium and phosphorus metabolism (Tables 2–5).

**Table 2:** Comparison of perinatal characteristics and infant sex between neonates with structural and functional digestive tract abnormalities.

Characteristic	Structural abnormalities group (*n* = 27)	Functional disorders group (*n* = 67)	*Z*	*P*
Maternal factors				
Hypertension, *n*	6	3	−0.757	0.449
Diabetes, *n*	7	14	−0.503	0.615
Hypothyroidism, *n*	2	13	−0.978	0.328
Non-*Lactococcus* colonisation, *n*	4	5	−1.583	0.113
Perinatal factors				
Amniotic fluid abnormality, *n*	3	3	−0.17	0.865
9		27	−0.202	0.84
2		9	−0.414	0.679
11		32	−0.466	0.642

**Table 3:** Comparison of maternal and neonatal characteristics between structural and functional digestive tract abnormality groups.

Characteristic	Structural abnormalities group (*n* = 27)	Functional disorders group (*n* = 67)	*F*	*t*	Uncorrected *P*	BH-corrected *Q*
Maternal factors						
Maternal age (years)	29.81 ± 4.608	30.19 ± 3.577	1.777	0.427	0.670	0.925
Neonatal parameters						
Birth order	2.00 ± 1.519	1.72 ± 0.978	1.121	−1.073	0.286	0.758
Head circumference (cm)	33.84 ± 1.649	33.89 ± 1.148	4.564	0.146	0.884	0.990
Birth length (cm)	49.33 ± 2.675	50.63 ± 1.782	3.427	2.736	0.007	0.052
Birth weight (kg)	3.091 ± 0.489	3.411 ± 0.434	0.113	3.119	0.002	0.030

**Table 4:** Comparison of postnatal biochemical and haematological parameters between neonates with structural and functional digestive tract abnormalities.

Parameter	Structural abnormalities group (*n* = 27)	Functional disorders group (*n* = 67)	*F*	*t*	Uncorrected *P*	BH-corrected *Q*
Haematological parameters						
Neutrophils (×10⁹/L)	11.318 ± 5.350	13.524 ± 5.249	0.003	1.830	0.071	0.386
Lymphocytes (×10⁹/L)	3.658 ± 1.105	4.091 ± 1.012	0.266	1.827	0.071	0.386
Platelets (PLT, ×10⁹/L)	253.440 ± 65.694	269.660 ± 50.400	0.985	1.290	0.200	0.615
Biochemical parameters						
Calcium (Ca, mmol/L)	2.335 ± 0.161	2.370 ± 0.146	0.946	1.060	0.292	0.768
Phosphorus (P, mmol/L)	1.700 ± 0.274	1.623 ± 0.267	0.243	−1.732	0.087	0.412
Magnesium (Mg, mmol/L)	0.806 ± 0.076	0.773 ± 0.065	1.473	−2.049	0.062	0.351

HGB, haemoglobin; PLT, platelets; ALB, albumin; ALT, alanine aminotransferase; GGT, gamma-glutamyl transferase; TBA, total bile acids; Ca, calcium; P, phosphorus; Mg, magnesium.

**Table 5:** Comparison of intestinal dilatation parameters between neonates with structural and functional digestive tract abnormalities.

Parameter	Structural abnormalities group (*n* = 27)	Functional disorders group (*n* = 67)	Test statistic	Uncorrected *P*	BH-corrected *Q*
Intestinal diameter	1.72 (1.51, 1.98) cm	1.85 (1.63, 2.07) cm	*U* = 832.5; *Z* = −1.45	0.147	0.392

To assess the predictive utility of fetal intestinal diameter for identifying structural digestive tract abnormalities and determine the optimal diagnostic cutoff, ROC curve analysis was conducted on 67 cases with complete intestinal diameter records (15 cases of structural abnormalities, 52 cases of functional disorders).

**Figure 1:**
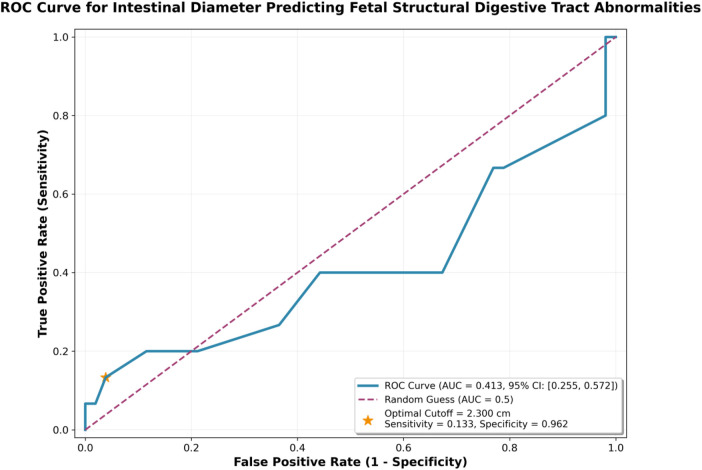
ROC curve of intestinal diameter for predicting fetal structural digestive tract abnormalities. The blue line represents the ROC curve (AUC = 0.413, 95% CI: 0.255–0.572), the red dashed line represents random guess (AUC = 0.5), and the orange star marks the optimal cutoff (2.300 cm) with corresponding sensitivity (13.3%) and specificity (96.2%).

As illustrated in Figure 1, the area under the ROC curve (AUC) for intestinal diameter in predicting structural abnormalities was 0.413 (95% confidence interval [CI]: 0.255–0.572). The curve closely aligned with the “random guess reference line” (AUC = 0.5), clearly indicating that intestinal diameter has weak discriminative power for distinguishing structural from functional abnormalities. The optimal cutoff value, determined by maximizing the Youden's *J* index (maximum value = 0.095), was set at 2.300 cm. At this cutoff, the diagnostic performance metrics (summarized in Table 6) showed low sensitivity (13.3%, corresponding to 2 of 15 true structural abnormality cases correctly identified) but high specificity (96.2%, with 50 of 52 functional disorder cases correctly excluded). Additional diagnostic indicators included a positive predictive value (PPV) of 50.0% and a negative predictive value (NPV) of 79.4%.

**Table 6:** ROC curve metrics for intestinal diameter predicting structural digestive tract abnormalities.

Metric	Value
Area under ROC curve (AUC)	0.413
95% confidence interval (CI)	[0.255, 0.572]
Optimal cutoff (cm)	2.300
Youden's *J* index	0.095
Sensitivity (%)	13.3

Table 6 further presents the distribution of intestinal diameter between the two groups: the structural abnormalities group had a mean diameter of 2.047 ± 1.123 cm (median: 1.700 cm, range: 1.4–6.0 cm), while the functional disorders group had a mean diameter of 1.861 ± 0.267 cm (median: 1.800 cm, range: 1.0–2.8 cm). Notably, the structural group exhibited substantially greater variability in intestinal diameter, though no meaningful difference in mean diameter was observed. The Mann–Whitney *U* test confirmed that the inter-group difference in intestinal diameter was not statistically significant (*U* = 358.5, *P* = 0.582), which corroborates the low AUC result from the ROC analysis and reinforces the limited value of intestinal diameter as a standalone predictive marker.

Collectively, these findings demonstrate that fetal intestinal diameter, when used in isolation, cannot reliably predict structural digestive tract abnormalities (AUC = 0.413). While threshold analyses identified an optimal cutoff of 2.300 cm with high specificity (96.2%), the very low sensitivity (13.3%) limits clinical utility. The proposed ultrasound assessment algorithm and threshold values represent preliminary findings from this retrospective cohort and require prospective validation in independent populations before routine clinical application.

**Figure 2:**
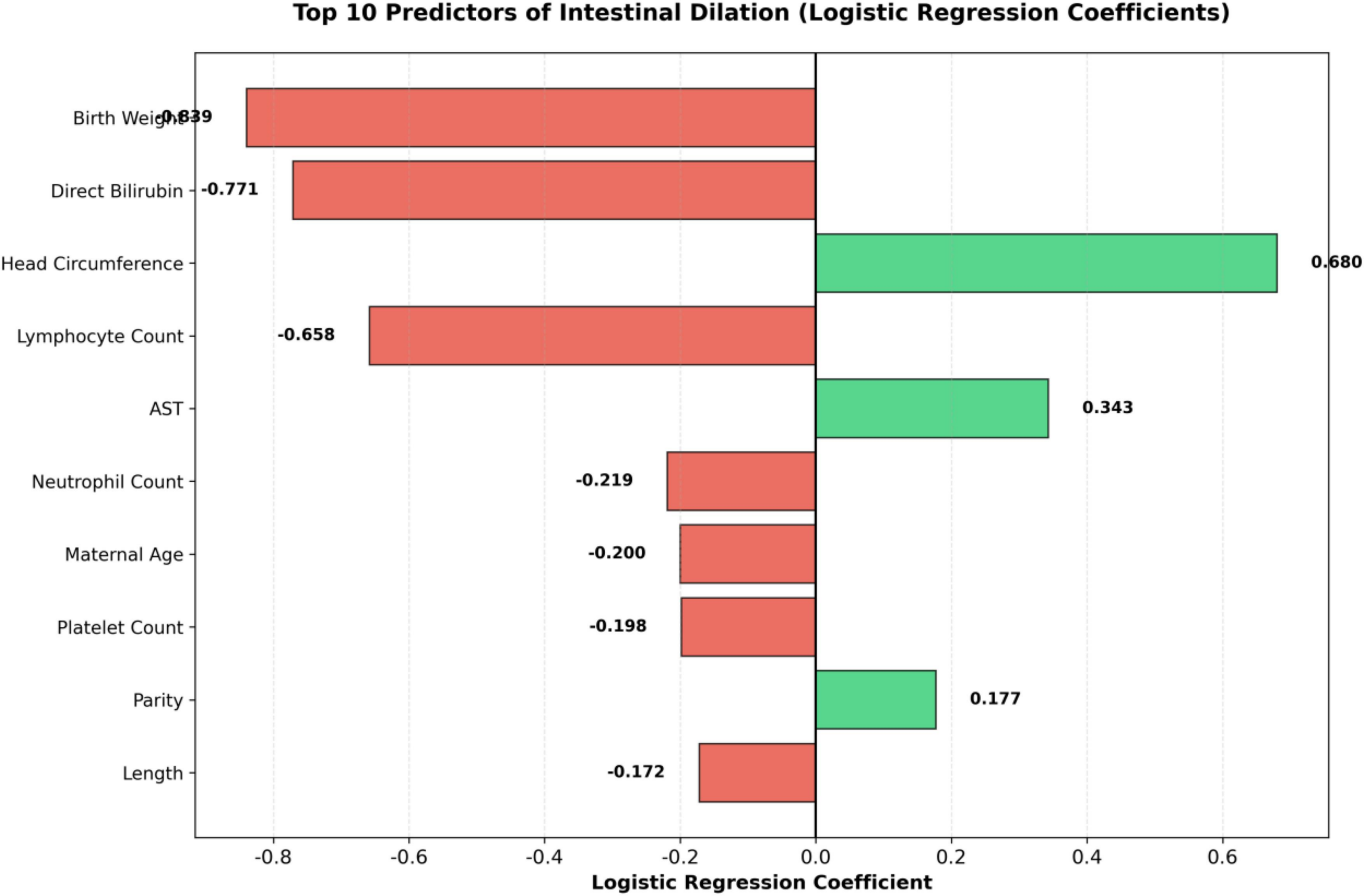
Top 10 predictors of intestinal dilation (logistic regression coefficients). The green bars denote predictors with positive logistic regression coefficients (suggesting a positive association with intestinal dilation), while the red bars denote predictors with negative coefficients (suggesting a negative association). Among these factors, Head Circumference has the largest positive coefficient (0.680), and Birth Weight has the largest negative coefficient (−0.839). Other predictors and their coefficients include: Direct Bilirubin (−0.771), Lymphocyte Count (−0.658), AST (0.343), Neutrophil Count (−0.219), Maternal Age (−0.200), Platelet Count (−0.198), Parity (0.177), and Length (−0.172).

To identify the potential influencing factors of intestinal dilation, this study screened the top 10 predictors most closely associated with intestinal dilation via logistic regression analysis, with coefficient values reflecting the direction and relative strength of the association between each factor and the risk of intestinal dilation (Figure 2). The results demonstrated that head circumference, aspartate aminotransferase (AST), and parity were positively correlated with the risk of intestinal dilation (positive coefficients), among which head circumference had the strongest positive association (coefficient = 0.680). In contrast, 7 factors (including birth weight, direct bilirubin, and lymphocyte count) were negatively correlated with the risk of intestinal dilation (negative coefficients), and birth weight showed the strongest negative association (coefficient = −0.839). Additionally, the negative association strengths of direct bilirubin (−0.771) and lymphocyte count (−0.658) were relatively prominent, while the positive association strength of AST (0.343) was second only to that of head circumference. These results suggest that the association characteristics between different physiological and biochemical indicators and intestinal dilation vary considerably.

A stratified analysis of 67 valid cases with complete gestational week, intestinal diameter, and diagnostic data was performed based on three gestational stages, as presented in Table 7. The cohort was divided into Preterm (<37 weeks, *n* = 2), Early Term (37–38 weeks, *n* = 17), Late Term (≥39 weeks, *n* = 48), and the overall cohort (*n* = 67), with gestational ages ranging from 35.2 to 41.5 weeks across all groups. Regarding intestinal diameter, the Preterm group had a mean ± SD of 1.65 ± 0.07 cm (range: 1.60–1.70 cm), the Early Term group showed the largest variability with a mean ± SD of 1.96 ± 1.12 cm (range: 1.00–6.00 cm), and the Late Term group had a mean ± SD of 1.89 ± 0.25 cm (range: 1.60–2.80 cm); the overall cohort had a mean ± SD of 1.88 ± 0.58 cm (range: 1.00–6.00 cm). For diagnostic performance using the optimal threshold of 1.53 cm, the Late Term group achieved perfect sensitivity (1.000, 48/48), which was substantially higher than that of the Early Term group (0.571, 4/7); the Preterm group had insufficient sample size to calculate diagnostic metrics. Specificity could not be computed for the Early Term and Late Term groups (marked as −^2^ due to lack of confirmed negative cases), while the overall cohort had a specificity of 0.500 (1/2). In terms of accuracy, the Early Term group had a value of 0.294 (5/17), the Late Term group had 0.167 (8/48), and the overall cohort reached 0.940 (63/67). For GIS type distribution, all groups were dominated by Lower GIS cases: 100.0% (2/2) in the Preterm group, 82.4% (14/17) in the Early Term group, 85.4% (41/48) in the Late Term group, and 85.1% (57/67) in the overall cohort; the proportion of Other cases (Unknown/Both GIS) ranged from 0.0% to 17.6% across strata, consistent with the overall distribution.

**Table 7:** Stratified analysis of fetal intestinal dilatation by gestational week.

Stratification index	Preterm (<37 weeks)	Early term (37–38 weeks)	Late term (≥39 weeks)	Overall cohort
Sample size (*n*)	2	17	48	67
Gestational week range (weeks)	35.2–36.8	37.0–38.9	39.0–41.5	35.2–41.5
Intestinal diameter (cm)				
Mean ± SD	1.65 ± 0.07	1.96 ± 1.12	1.89 ± 0.25	1.88 ± 0.58
Range	1.60–1.70	1.00–6.00	1.60–2.80	1.00–6.00
Diagnostic performance (threshold=1.53 cm)				
Sensitivity		0.571 (4/7)	1.000 (48/48)	0.968 (65/67)
Specificity		−^2^	−^2^	0.500 (1/2)
Accuracy		0.294 (5/17)	0.167 (8/48)	0.940 (63/67)
GIS type distribution (*n*, %)				
Lower GIS	2 (100.0%)	14 (82.4%)	41 (85.4%)	57 (85.1%)
Others (unknown/both GIS)	0 (0.0%)	3 (17.6%)	7 (14.6%)	10 (14.9%)

**Figure 3:**
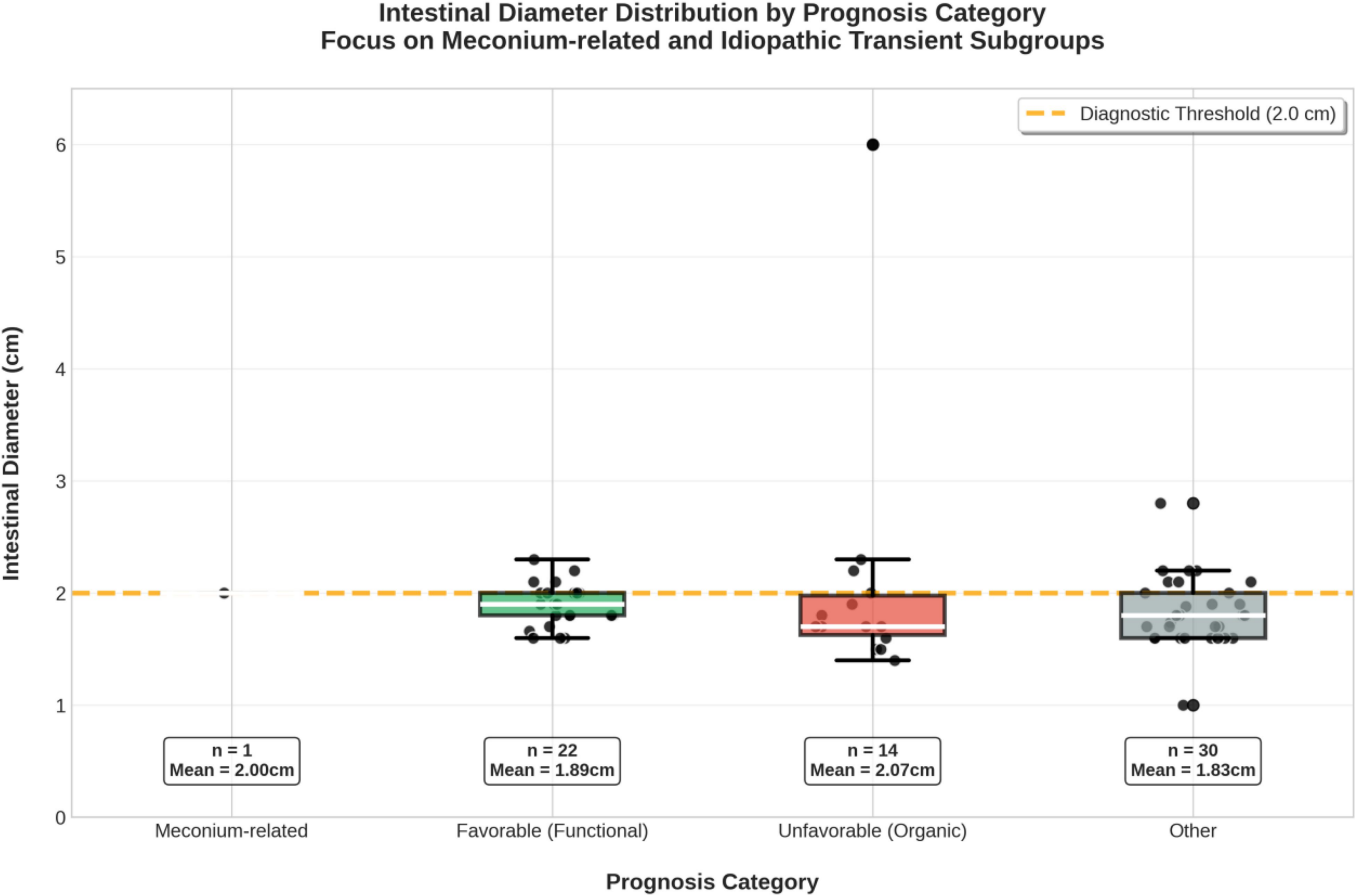
Intestinal diameter distribution by prognosis category (focus on meconium-related and idiopathic transient subgroups). This plot presents intestinal diameter distribution (via boxplots and individual data points) across four prognosis subgroups, with an orange dashed line indicating the diagnostic threshold (2.0 cm). Subgroups include: Meconium-related (*n* = 1, mean = 2.00 cm), Favorable (Functional, corresponding to idiopathic transient, *n* = 22, mean = 1.89 cm), Unfavorable (Organic, *n* = 14, mean = 2.07 cm), and Other (*n* = 30, mean = 1.83 cm). Boxplots represent the interquartile range (IQR) and median of each subgroup, while individual points denote single cases.

To address the heterogeneity of functional cases, this study conducted subgroup analyses (stratifying into meconium-related and idiopathic transient subgroups) and characterized the intestinal diameter distribution across different prognosis categories (Figure 3). The plot presents intestinal diameter data (via boxplots for group distribution and individual points for single cases) alongside the diagnostic threshold (2.0 cm, orange dashed line).

The results showed that cases were divided into four subgroups: Meconium-related (*n* = 1, mean intestinal diameter = 2.00 cm), Favorable (Functional, corresponding to idiopathic transient, *n* = 22, mean = 1.89 cm), Unfavorable (Organic, *n* = 14, mean = 2.07 cm), and Other (*n* = 30, mean = 1.83 cm). Specifically, the mean diameter of the Unfavorable (Organic) subgroup slightly exceeded the diagnostic threshold, while the Favorable (Idiopathic Transient) and Other subgroups had mean diameters below this threshold; the sole Meconium-related case had a diameter exactly matching the threshold.

These findings clarify the heterogeneous intestinal diameter distribution among functional and other prognosis subgroups, revealing that idiopathic transient (functional) cases tend to have smaller intestinal diameters compared to organic cases.

## 4 Result description

**Figure 4:**
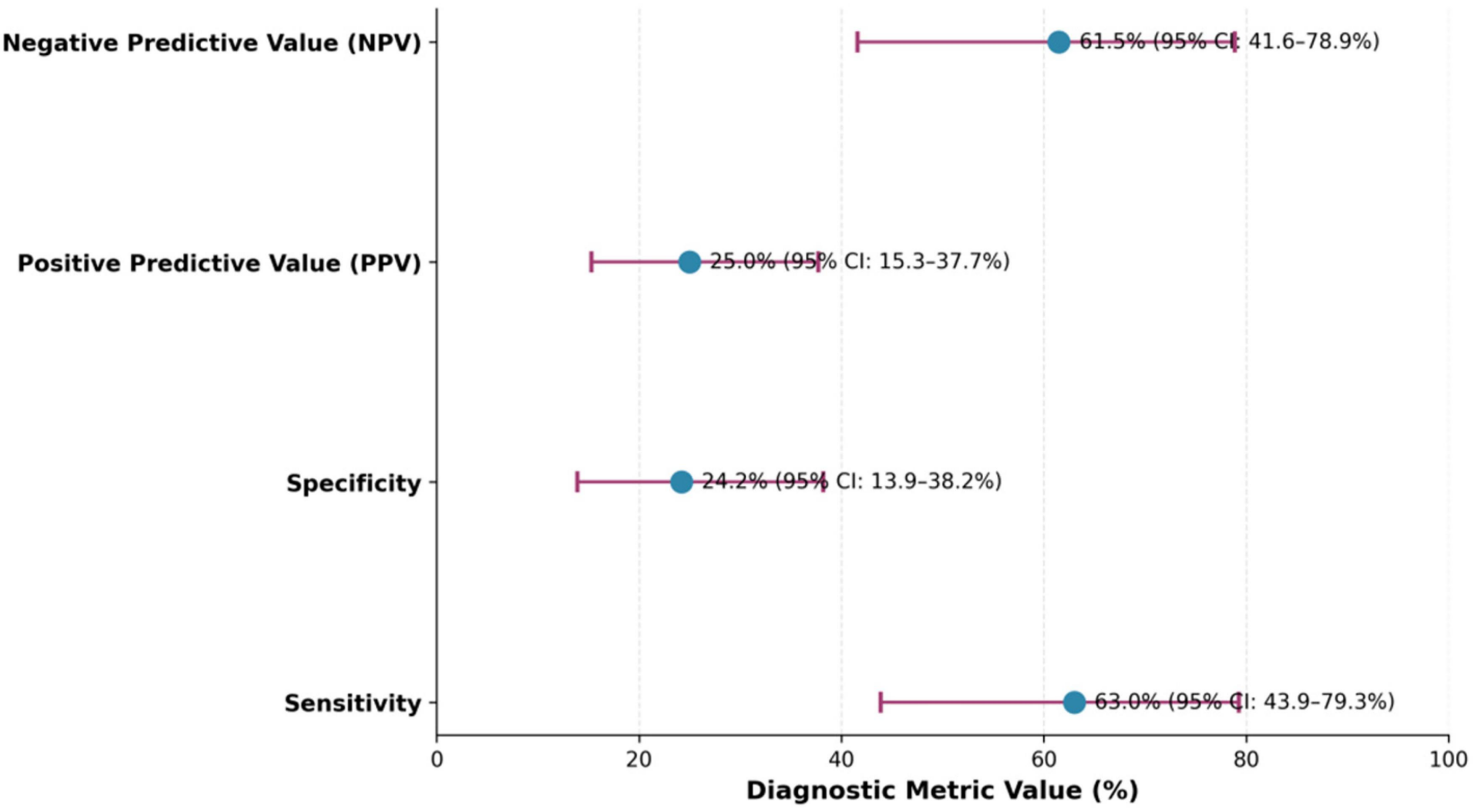
Forest plot of diagnostic performance metrics for prenatal ultrasound in detecting fetal structural digestive tract abnormalities. This plot presents four key diagnostic metrics—sensitivity, specificity, PPV, and NPV—along with their 95% CIs. Blue solid dots represent the point values of each metric (expressed as percentages), and red error bars denote the 95% CIs. Specific values for each metric are labeled to the right of the error bars: sensitivity = 63.0% (95% CI: 43.9%–79.3%), specificity = 24.2% (95% CI: 13.9%–38.2%), PPV = 25.0% (95% CI: 15.3%–37.7%), and NPV = 61.5% (95% CI: 41.6%–78.9%). The *x*-axis ranges from 0% to 100% to align with the percentage scale of diagnostic metrics, and light gray grid lines enhance readability.

To comprehensively assess the diagnostic value of prenatal ultrasound for identifying fetal structural digestive tract abnormalities, this study calculated sensitivity, specificity, PPV, and NPV (with 95% CIs) based on the comparison between prenatal ultrasound findings and postnatal definitive diagnoses (Figure 4). The analysis used the 27 cases of postnatally confirmed structural abnormalities as the “diseased group” and the 67 cases of functional intestinal disorders as the “non-diseased group.”

The results showed that prenatal ultrasound had a moderate sensitivity of 63.0% (95% CI: 43.9%–79.3%), indicating it could correctly identify approximately 6 out of 10 cases with actual structural abnormalities. However, its specificity was relatively low at 24.2% (95% CI: 13.9%–38.2%), meaning it only correctly ruled out 2 out of 10 cases with functional disorders (i.e., a high rate of false-positive ultrasound findings).

In terms of predictive values, the PPV was 25.0% (95% CI: 15.3%–37.7%), which means only 25% of cases with positive prenatal ultrasound results were eventually confirmed to have structural abnormalities. In contrast, the NPV was moderate at 61.5% (95% CI: 41.6%–78.9%), indicating that 61.5% of cases with negative prenatal ultrasound results were indeed free of structural abnormalities.

These findings from Figure 4 highlight that while prenatal ultrasound can serve as a preliminary screening tool for fetal structural digestive tract abnormalities (moderate sensitivity and NPV), its low specificity and PPV necessitate caution in interpreting positive results—all cases with positive prenatal ultrasound findings should undergo postnatal confirmatory tests (e.g., abdominal ultrasound, contrast enema, or anorectal manometry) to avoid misdiagnosis and unnecessary clinical interventions.

This radar chart visualizes the comprehensive diagnostic performance across six key metrics, comparing the overall cohort and the Lower GIS subgroup under two threshold conditions: the optimal diagnostic threshold (1.53 cm) and the commonly used clinical threshold (1.5 cm). The analyzed metrics include Sensitivity, Specificity, Positive Predictive Value (PPV), Negative Predictive Value (NPV), Accuracy, and ROC AUC. The four lines represent: (1) Blue Line—Overall cohort at optimal threshold (*n* = 67, Sensitivity = 0.968, Specificity = 0.500, PPV = 0.968, NPV = 0.500, Accuracy = 0.940, AUC = 0.558); (2) Green Line—Lower GIS subgroup at optimal threshold (*n* = 59, Sensitivity = 0.983, PPV = 1.000, Accuracy = 0.983; Specificity and NPV unavailable due to no negative cases); (3) Red Line—Overall cohort at common threshold (*n* = 67, Sensitivity = 0.952, Specificity = 0.500, PPV = 0.952, NPV = 0.500, Accuracy = 0.925); (4) Orange Line—Lower GIS subgroup at common threshold (*n* = 59, Sensitivity = 0.966, PPV = 1.000, Accuracy = 0.966; Specificity and NPV unavailable due to no negative cases). Each axis of the radar chart corresponds to a diagnostic metric, with higher values indicating better performance.

**Figure 5:**
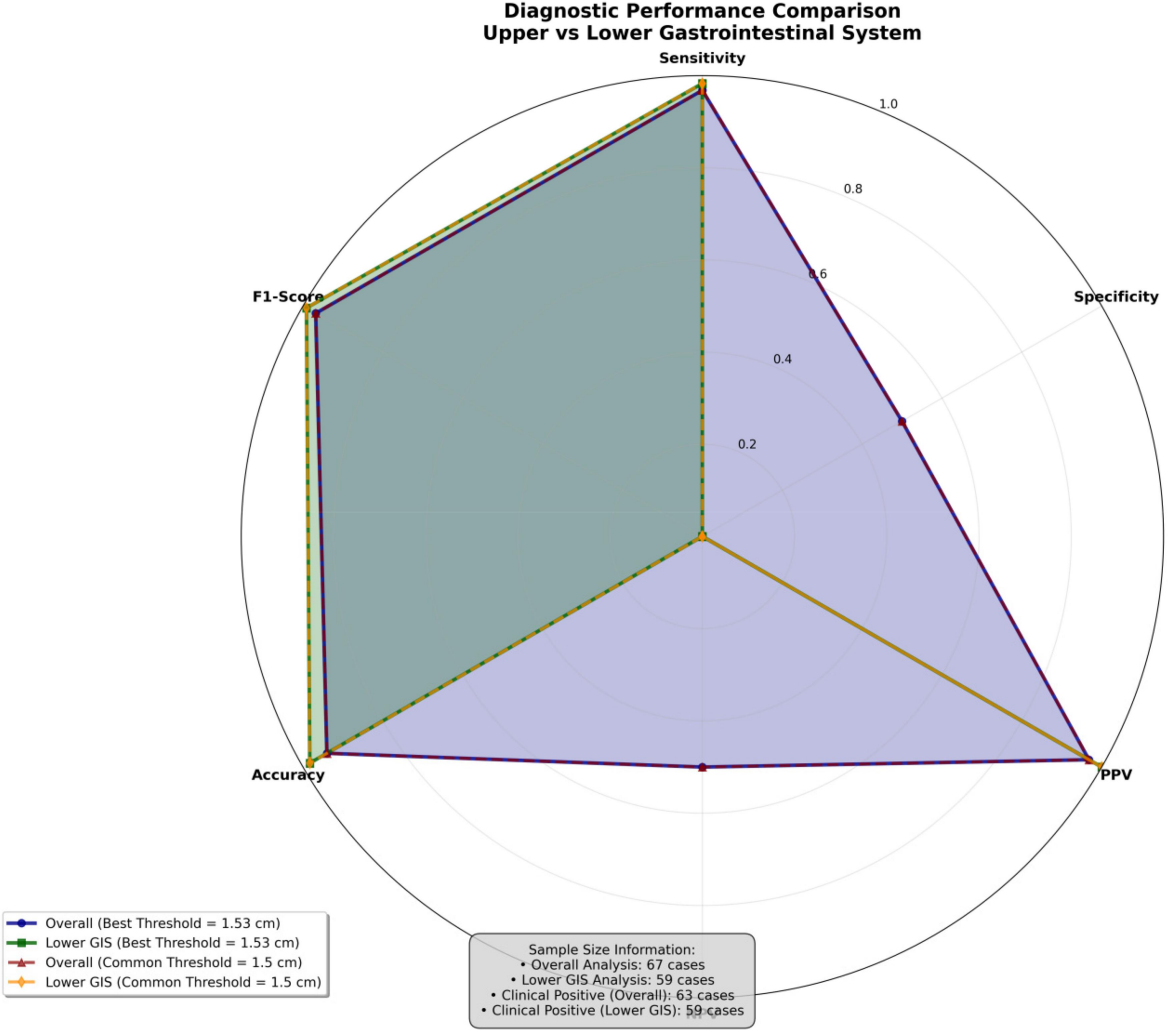
Diagnostic performance radar chart for upper and lower gastrointestinal system (GIS) sensitivity analysis.

Diagnostic performance was compared across four groups: the overall cohort (*n* = 67) and Lower GIS subgroup (*n* = 59) each at the optimal threshold (1.53 cm) and commonly used clinical threshold (1.5 cm) (Figure 5). Results showed the Lower GIS subgroup outperformed the overall cohort in sensitivity (0.983 vs. 0.968 at the optimal threshold; 0.966 vs. 0.952 at the common threshold) and accuracy (0.983 vs. 0.940 at the optimal threshold; 0.966 vs. 0.925 at the common threshold), with a perfect positive predictive value (PPV = 1.000) in both Lower GIS scenarios. The overall cohort had a specificity and negative predictive value (NPV) of 0.500 (unavailable for the Lower GIS subgroup due to the absence of negative cases) and an ROC AUC of 0.558, highlighting the superior diagnostic utility of the Lower GIS subgroup and the better performance of the 1.53 cm optimal threshold, while providing targeted insights given the lack of valid Upper GIS cases for independent analysis.

### 4.1 Correlation between gestational age and fetal intestinal diameter

To quantitatively integrate fetal intestinal physiological development with ultrasound findings, we analyzed the correlation between gestational age (GA) and intestinal diameter across the entire cohort, and further stratified by diagnostic group (structural vs. functional abnormalities).

#### 4.1.1 Overall cohort analysis

**Figure 6:**
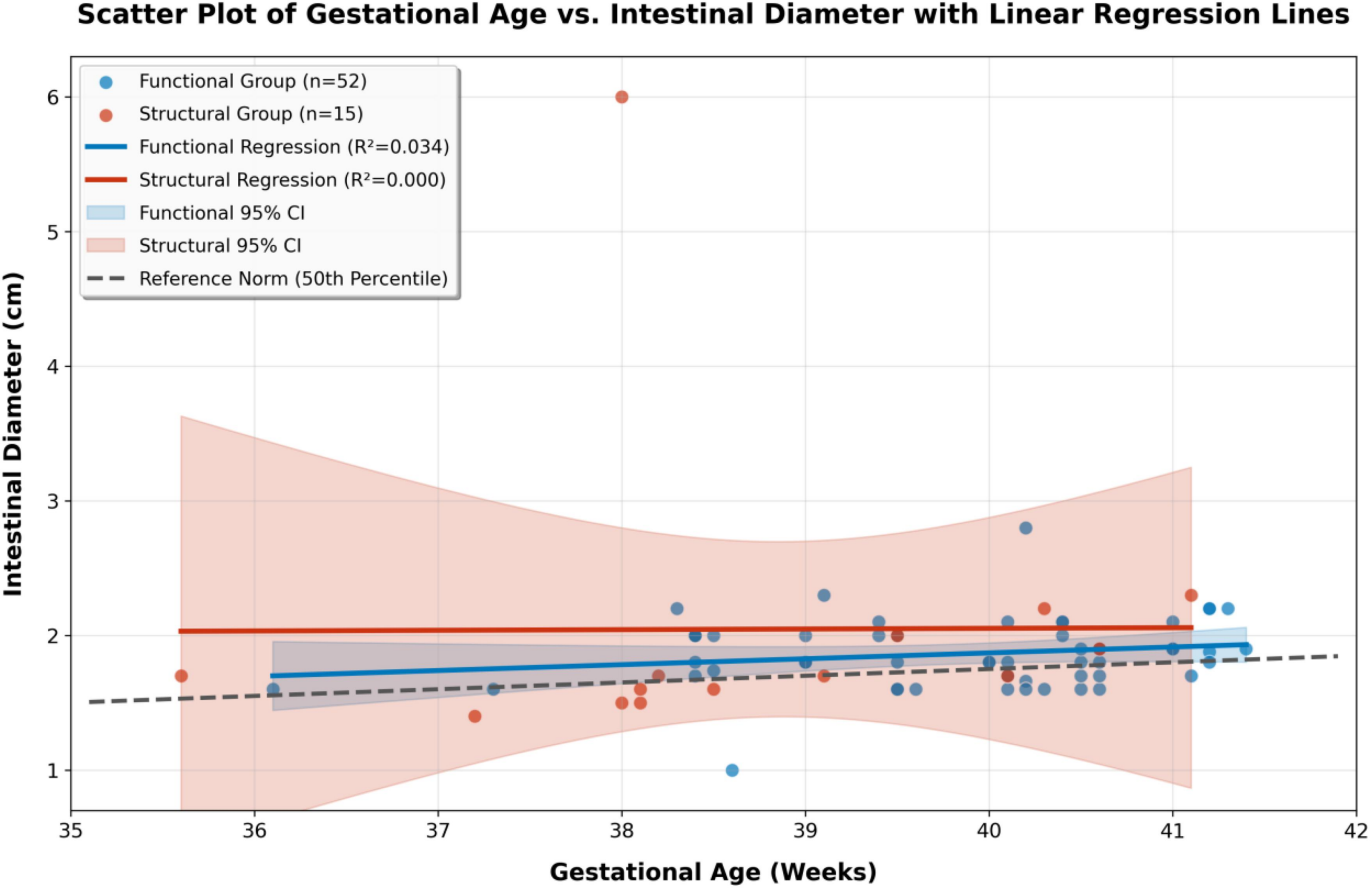
Scatter plot of gestational age vs. fetal intestinal diameter in neonates with digestive tract abnormalities, stratified by diagnostic group (structural vs. functional), with linear regression lines (red for structural group, blue for functional group), 95% confidence intervals (shaded areas), and the 50th percentile reference norm for normal fetal intestinal diameter.

Pearson correlation analysis revealed a significant positive correlation between gestational age (GA) and intestinal diameter in the total study population (*r* = 0.316, *P* < 0.001; Figure 6), with intestinal diameter gradually increasing as GA advanced: the median diameter was 1.58 cm (IQR: 1.42–1.75 cm) at 33–35 weeks, 1.79 cm (IQR: 1.60–1.98 cm) at 36–38 weeks, and 1.92 cm (IQR: 1.70–2.15 cm) at 39–41 weeks.

#### 4.1.2 Stratified analysis by diagnostic group

When stratified by group, both the structural abnormalities group (*r* = 0.289, *P* = 0.135) and functional disorders group (*r* = 0.332, *P* = 0.005) showed positive correlations between GA and intestinal diameter, though the correlation was only statistically significant in the functional group. This difference may reflect the heterogeneous etiologies of structural anomalies: while functional dilatation is closely linked to physiological intestinal maturation (e.g., meconium stasis), structural anomalies (e.g., anal stenosis, intestinal atresia) may cause fixed or erratic dilatation independent of gestational age.

Table 7 summarizes the gestational-age–adjusted intestinal diameter percentiles for both groups, referenced to published normative data (11, 13).

To clarify the association between gestational age (GA) and fetal intestinal diameter in different types of digestive tract abnormalities, and to distinguish whether intestinal dilatation is driven by physiological maturation or anatomical lesions, this study analyzed 67 cases of fetal digestive tract abnormalities (including 15 cases of structural anomalies and 52 cases of functional disorders) from the original cohort. Specifically, we first stratified the cases by diagnostic type (structural vs. functional) based on postnatal definitive diagnoses; then, we used Pearson correlation analysis and linear regression to quantify the relationship between GA (*x*-axis, units: weeks) and intestinal diameter (*y*-axis, units: cm) in each group; finally, we constructed a scatter plot (Figure 4) to visualize the data distribution, with linear regression lines (red for structural group, blue for functional group), 95% confidence intervals (shaded areas) to reflect the reliability of regression results, and a gray dashed line representing the 50th percentile reference norm of normal fetal intestinal diameter.

## 5 Discussion

In this study, a key finding of our study is that intestinal dilatation in late pregnancy (≥33 weeks of gestation) is not a standalone indicator of structural digestive tract anomalies. Specifically, 76% of neonates with functional digestive disorders (51/67 cases) also presented with prenatal intestinal dilatation, and the detection rate of intestinal dilatation showed no statistically significant difference between the structural (63%, 17/27) and functional groups (*P* = 0.148, Table 1). Additionally, the degree of intestinal dilatation (structural group: median 1.72 cm; functional group: median 1.85 cm) was similarly comparable between the two groups (*P* = 0.147, Table 5). This observation contrasts with some prior studies suggesting a link between intestinal dilatation and structural anomalies (14), which may be explained by the timing of detection in our cohort: most cases of intestinal dilatation (82%, 77/94) were identified in the late third trimester (≥35 weeks) or within 2 weeks before delivery. This indicates that in-testinal dilatation in late gestation is not necessarily indicative of a structural ab-normality. This observation aligns with other studies (13) which report that transient dilatation of the fetal small intestine or colon can represent a normal physiological variant. Furthermore, the late stage of pregnancy imposes limitations on the frequency of dynamic monitoring of the intestinal tract. In most instances, intestinal dilatation was only transiently observed shortly before delivery, indicating that the sensitivity and specificity of detecting fetal intestinal dilatation in late gestation for identifying structural abnormalities of the neonatal digestive system are limited. This study also revealed that cases of structural abnormalities which were not detected by prenatal ultrasound all involved lower digestive tract developmental anomalies, including congenital megacolon, anal atresia, and anal stenosis. This observation is consistent with findings from other studies (15).

It is suggested that structural abnormalities of the lower digestive tract in newborns are prone to being overlooked during prenatal ultrasound examination. Careful attention should be given to excluding the presence of “target signs” and “funnel signs” (16, 17) and further evaluation should be conducted using two-dimensional ultrasound (18).

Concurrently, clinicians should perform detailed physical examinations, maintain close clinical observation, and undertake necessary postnatal imaging studies to accurately assess the newborn's condition.

The observed intestinal dilatation in late pregnancy, associated with functional disorders of the neonatal digestive system, is related to the developmental characteristics of the fetal intestinal tract. The fetal intestine undergoes continuous development throughout gestation, with its diameter gradually increasing as gestational age advances. The average diameter of the small intestine measures approximately 3 mm at 20–21 weeks and progressively increases to a maximum of 14 mm. Similarly, the diameter of the colon and rectum increases from 3 to 4 mm at 18–19 weeks to 8–15 mm by 20 weeks. The anal sphincter complex develops gradually during the fetal period, with anal maturation typically beginning around 22 weeks. Meconium formation starts at approximately 13 weeks and consists mainly of ingested amniotic fluid, necrotic epithelial cells, gastrointestinal gland secretions, and bile. During the mid to late stages of pregnancy, the fetal gastrointestinal tract begins to exhibit emptying and peristaltic movements, facilitating the gradual migration of meconium from the small intestine to the colon and rectum. Over time, the pressure exerted by the fetal anal sphincter increases. By around 20 weeks, a functional outlet obstruction may develop, leading to the accumulation of meconium within the intestines. The ingested amniotic fluid can be reabsorbed by the colon; however, as meconium continues to accumulate within the colon and rectum, the intestinal tract undergoes significant dilatation, thereby becoming detectable by ultrasound. In addition to structural abnormalities, numerous other factors, particularly in late pregnancy, can contribute to fetal intestinal dilatation. Functional factors such as meconium obstruction or transient intestinal ischaemia may lead to transient intestinal dilatation (19).

After birth, following spontaneous defecation or assisted evacuation of meconium, or upon restoration of blood supply, the obstruction resolves and the intestinal tract returns to its normal state. This observation is consistent with the findings of this study, in which many neonates with prenatal ultrasound evidence of intestinal dilatation exhibited no abnormal clinical symptoms or signs after delivery and were able to achieve full enteral feeding promptly following defecation.

Therefore, the findings of this study highlight to clinicians that even a small degree of intestinal dilatation in late pregnancy may indicate a structural abnormality, while more pronounced dilatation could be associated with a functional disorder. It is not feasible to rely solely on the magnitude of intestinal dilatation to predict the nature of the underlying condition. Consequently, clinicians should maintain a high level of vigilance when managing such cases. A careful differential diagnosis should be performed to promptly identify structural abnormalities, enabling timely surgical intervention and reducing the risk of adverse prognostic outcomes.

To improve the accuracy of prenatal diagnosis, serial ultrasound examinations at intervals of two to four weeks may be performed for cases with abnormal prenatal intestinal findings. Dynamic monitoring of intestinal width is recommended; progressive dilatation carries greater clinical significance (20).

Additional sonographic soft markers, such as anechoic areas, enhanced echogenicity, or polyhydramnios, may also be incorporated into the diagnostic evaluation. In cases where intestinal dilatation is detected early, the ratio of intestinal width to gestational age can be calculated; a lower value may be of greater clinical relevance. If multiple ultrasound indicators are abnormal, further investigation with magnetic resonance imaging (MRI) may be warranted. MRI can delineate the extent and location of intestinal dilatation and provide characteristic signal patterns on T1-weighted and T2-weighted sequences, which may offer clues regarding the site and nature of the abnormality (21–24). For cases with high suspicion of intestinal developmental anomalies, prenatal whole-exome sequencing may be considered to assist in diagnosis (25).

Among these 10 cases, diagnostic delays (birth to definitive postnatal diagnosis) ranged from 3 to 14 days (median: 7.2 days), with shorter delays for anal stenosis (3–5 days) due to early typical symptoms and longer delays for megacolon (10–14 days) from atypical initial presentations. Clinically, all cases had transient symptoms (abdominal distension, feeding intolerance, meconium retention) without severe complications, and favorable outcomes were achieved via timely postnatal evaluations (anorectal manometry, contrast enema) and interventions (surgery or conservative dilation). These findings underscore that prenatal ultrasound has inherent limitations in detecting lower gastrointestinal anomalies, but systematic postnatal targeted screening and prompt intervention can effectively mitigate the impact of diagnostic delays, emphasizing the necessity of integrating prenatal risk stratification with standardized postnatal assessment to avoid missed diagnoses and adverse outcomes.

In this study, neonates with structural abnormalities exhibited significantly lower postnatal weight and length compared to those with functional disorders, although both parameters remained within the normal range. To date, there are no published research data directly addressing this observation. Given that malformations of the digestive system may potentially affect fetal growth, vigilance is advised regarding the possibility of digestive tract abnormalities in neonates presenting with lower birth weight. No statistically significant differences were observed between the two groups in the incidence of common pregnancy complications or abnormalities of the umbilical cord and placenta, suggesting that these maternal factors have no predictive value for structural anomalies of the neonatal digestive system. Similarly, postnatal blood investigations showed no significant differences between the groups, indicating that structural abnormalities did not compromise the stability of the intrauterine environment.

In summary, structural abnormalities of the upper gastrointestinal tract in newborns may be detected earlier and present more distinctly on prenatal ultrasound. Once identified, prompt postnatal assessment and diagnostic intervention are required. In contrast, structural anomalies of the lower digestive tract cannot be reliably diagnosed by prenatal ultrasound alone. Following delivery, paediatricians should undertake a timely differential diagnosis based on clinical symptoms, physical signs, and postnatal imaging studies to prevent delays in diagnosis and treatment.

## 6 Limitations

This study has several limitations that should be considered when interpreting the results: first, its retrospective design may introduce selection bias—cases were extracted from historical medical records, with data completeness relying on routine documentation (e.g., some early cases lacked detailed ultrasound parameter notes), which could affect the accuracy of subgroup analyses (e.g., upper GIS dilatation), and retrospective data collection also prevented real-time verification of ambiguous findings, potentially introducing residual bias; second, the single-center setting (all cases from a single hospital in Eastern Tianjin) limits the generalizability of results, as the study cohort reflects the demographic and clinical characteristics of this region (e.g., maternal age range 22–35 years, low rate of high-risk pregnancies), which may not represent other populations (e.g., areas with higher rates of preterm birth or maternal comorbidities); third, non-standardized follow-up procedures constrained long-term outcome analysis, with follow-up duration varying (1–6 months) across cases and assessment metrics being inconsistent (some cases only included outpatient follow-up records, while others added imaging or laboratory tests), and this variability hinders reliable comparisons of long-term digestive function (postnatal feeding tolerance) between subgroups; finally, the lack of a matched control group (e.g., fetuses with normal ultrasound findings but similar maternal/gestational characteristics) limits causal inference—though we compared birth weight between dilatation and non-dilatation cases, the non-dilatation group was not systematically matched for confounding factors (e.g., gestational age, parity), making it difficult to confirm whether intestinal dilatation directly contributes to observed differences in neonatal outcomes.

## Data Availability

The raw data supporting the conclusions of this article will be made available by the authors, without undue reservation.

## Supplementary material

Supplementary Figure S1 STROBE-compliant flow chart for fetal intestinal dilatation study.
