# Supplementary material for: Fetal intestinal dilatation and digestive tract abnormalities: an analysis of 94 cases from 16,090 deliveries in eastern Tianjin
Source: Front Pediatr. 2026 Jul 27;14:1862935. doi: 10.3389/fped.2026.1862935 (PMC13454390; doi:10.3389/fped.2026.1862935)
Supplement: Supplementary file 1 [file Supplementaryfile1.docx]

Figure S1. STROBE-Compliant Flow Chart for Fetal Intestinal Dilatation Study
